# A weighted non-negative matrix factorization approach to predict potential associations between drug and disease

**DOI:** 10.1186/s12967-022-03757-1

**Published:** 2022-12-03

**Authors:** Mei-Neng Wang, Xue-Jun Xie, Zhu-Hong You, De-Wu Ding, Leon Wong

**Affiliations:** 1 School of Mathematics and Computer Science, Yichun University, Yichun, 336000 Jiangxi China; 2 School of Computer Science, Northwestern Polytechnical University, Xi’an, 710072 China; 3 Xinjiang Technical Institutes of Physics and Chemistry, Chinese Academy of Sciences, Urumqi, 830011 China; 4 University of Chinese Academy of Sciences, Beijing, 100049 China

**Keywords:** Drug-disease association, Weighted nearest neighbor, Graph regularization, Non-negative matrix factorization

## Abstract

**Background:**

Associations of drugs with diseases provide important information for expediting drug development. Due to the number of known drug-disease associations is still insufficient, and considering that inferring associations between them through traditional in vitro experiments is time-consuming and costly. Therefore, more accurate and reliable computational methods urgent need to be developed to predict potential associations of drugs with diseases.

**Methods:**

In this study, we present the model called weighted graph regularized collaborative non-negative matrix factorization for drug-disease association prediction (WNMFDDA). More specifically, we first calculated the drug similarity and disease similarity based on the chemical structures of drugs and medical description information of diseases, respectively. Then, to extend the model to work for new drugs and diseases, weighted $K$ nearest neighbor was used as a preprocessing step to reconstruct the interaction score profiles of drugs with diseases. Finally, a graph regularized non-negative matrix factorization model was used to identify potential associations between drug and disease.

**Results:**

During the cross-validation process, WNMFDDA achieved the AUC values of 0.939 and 0.952 on Fdataset and Cdataset under ten-fold cross validation, respectively, which outperforms other competing prediction methods. Moreover, case studies for several drugs and diseases were carried out to further verify the predictive performance of WNMFDDA. As a result, 13(Doxorubicin), 13(Amiodarone), 12(Obesity) and 12(Asthma) of the top 15 corresponding candidate diseases or drugs were confirmed by existing databases.

**Conclusions:**

The experimental results adequately demonstrated that WNMFDDA is a very effective method for drug-disease association prediction. We believe that WNMFDDA is helpful for relevant biomedical researchers in follow-up studies.

## Background

In the past few decades, people have made remarkable progress in life sciences and genomics. However, the development of a new drug is still a high-risky, tremendously expensive and time-consuming process [1, 2]. On average, it takes about 15 years and costs more than $ 800 million to discover and bring a new drug to the market [3, 4]. Although tremendous investment in new drugs design and discovery, the number of new drugs authorized by the U.S. Food and Drug Administration (FDA) has remained low since the 1990s [5, 6]. About 90% new drugs designed for specific diseases fail the first phase of clinical trials, which means that new drugs design and discovery are becoming more and more costly [7]. In light of these challenges, repositioning of already commercialized drugs, which aims to identify and discover the new therapeutic uses for these drugs, is attracting strong increasing interests from the biomedical researchers and pharmaceutical companies [8]. Since existing drugs have been proven safe through various clinical trials, drug repositioning can lower risk, shorten the process of drug development, and are more likely to be approved by regulatory authorities [9]. Therefore, drug repositioning plays an important role in drug research and development. Nowadays, some existing drugs (e.g. Minoxidil, Thalidomide, Sildenafil) have been successfully repositioned in clinical trials, which have saved new drug development costs and created great economic value for related pharmaceutical companies [10]. For example, Minoxidil, originally commercialized to prevent high blood pressure, was repositioned to treat the androgenic alopecia; Thalidomide was marketed to use as a sedative, it was later repurposed as a treatment to insomnia and nausea [11, 12]. Compared with the development of a novel drug for specific indications, drug repositioning costs only about $ 300 million and can shorten the drug development cycle by more than half [10, 13]. To this end, more and more existing drugs are being repurposed to treat diseases other than those originally intended [14].

In fact, drug repositioning can be seen as identifying the associations between drug and disease. Although some associations of drugs with diseases have been verified in clinical trials, many of them are still undiscovered. In recent years, some computational approaches have been developed to infer associations between drug and disease for drug repositioning, such as semantic inference [1], network analysis [15], text mining [16] and machine learning [17], etc. For example, Napolitano et al. trained a multi-class Support Vector Machine (SVM) classifier based on drugs similarities to identify potential drug indications [18]. Gottlieb et al. constructed classification features by integrating disease similarities and drug similarities, and scored the new associations of drugs with diseases to predict novel therapeutic indications by implementing a logistic regression classification algorithm [19]. Based on the hypothesis that different diseases with similar treatments can be treated with similar drugs, Chiang et al. developed a “guilt-by-association” principle approach to infer potential relationships between drug and disease [20]. Yang et al. developed a causal network linking drug-target-pathway-gene-disease to calculate association scores of drugs with diseases. Based on known drug-disease associations, a probabilistic matrix factorization model is learned to classify drug–disease associations, and novel associations of drugs with diseases were predicted according to the calculated association scores and association types [21]. However, these methods fail to predict associations of novel drugs without any known related disease.

At present, with the generation of large-scale high-throughput biological data, researchers are increasingly concerned how to establish complex biomolecular interaction networks for predicting their associations. Martínez et al. have developed a novel model, DrugNet, to infer new treatments for diseases and novel therapeutic indications for drugs [22]. This method predicts drug-disease potential associations by prioritizing based on a heterogeneous network which was integrated biological information about drugs, targets and diseases. Wang et al. proposed three-layer heterogeneous network-based computational method named TL-HGBI, which performs drug repositioning by applying known drug-disease associations and drug, disease and target similarities [23]. Luo et al. presented a new prediction model MBiRW, which utilized Bi-Random walk algorithm to infer new drug indications based on the assumption that similar drugs tend to be associated with the different diseases that with similar treatments [24].

In fact, predicting novel indications for existing drugs can be considered as a recommendation system problem. Recently, recommendation system models have been used to predict associations between biomolecules (e.g. drug-target interactions, circRNA-disease associations) [25, 26]. Luo et al. developed a drug repositioning recommendation system (DRRS) to infer new indications for existing drugs, which used fast Singular Value Thresholding (SVT) algorithm to complete the association adjacency matrix of drug with disease [27]. Similar to finding missing interactions in an adjacency matrix, matrix factorization is well applied in collaborative filtering recommendation algorithms [28]. Recent studies have shown that matrix factorization technique has been successfully used in recommender system and link prediction for data representation [29, 30], especially in the field of bioinformatics [31–33]. Inspired by these, we can view the drug-disease association prediction problem as a recommender system task and used matrix factorization to predict.

In this paper, we propose a new computational method named WNMFDDA to infer the unknown associations of drugs with diseases, which is based on weighted graph regularized collaborative non-negative matrix factorization. Distinct from previous methods, graph Laplacian regularization is introduced to prevent overfitting, which can ensure close drugs or diseases are sufficiently close to each other in the corresponding latent feature space; Tikhonov (${L}_{2}$) is used to guarantee that the solution obtained from matrix factorization is smooth. In addition, in order to extend our model to work for new drugs (or new diseases) and reduce the impact of sparse associations on prediction performance, weighted $K$-nearest neighbor is utilized to rebuild the association adjacency matrix between drug and disease before performing matrix factorization. We carry out ten-fold cross validation to verify the performance of WNMFDDA and compared it with several classical models. The experimental results of cross validation show that WNMFDDA obtains better performance than other compared models. Case studies on drugs and diseases also demonstrate that our proposed approach is reliable in identifying drug-disease potential associations.

## Methods and materials

### Method overview

**Fig. 1:**
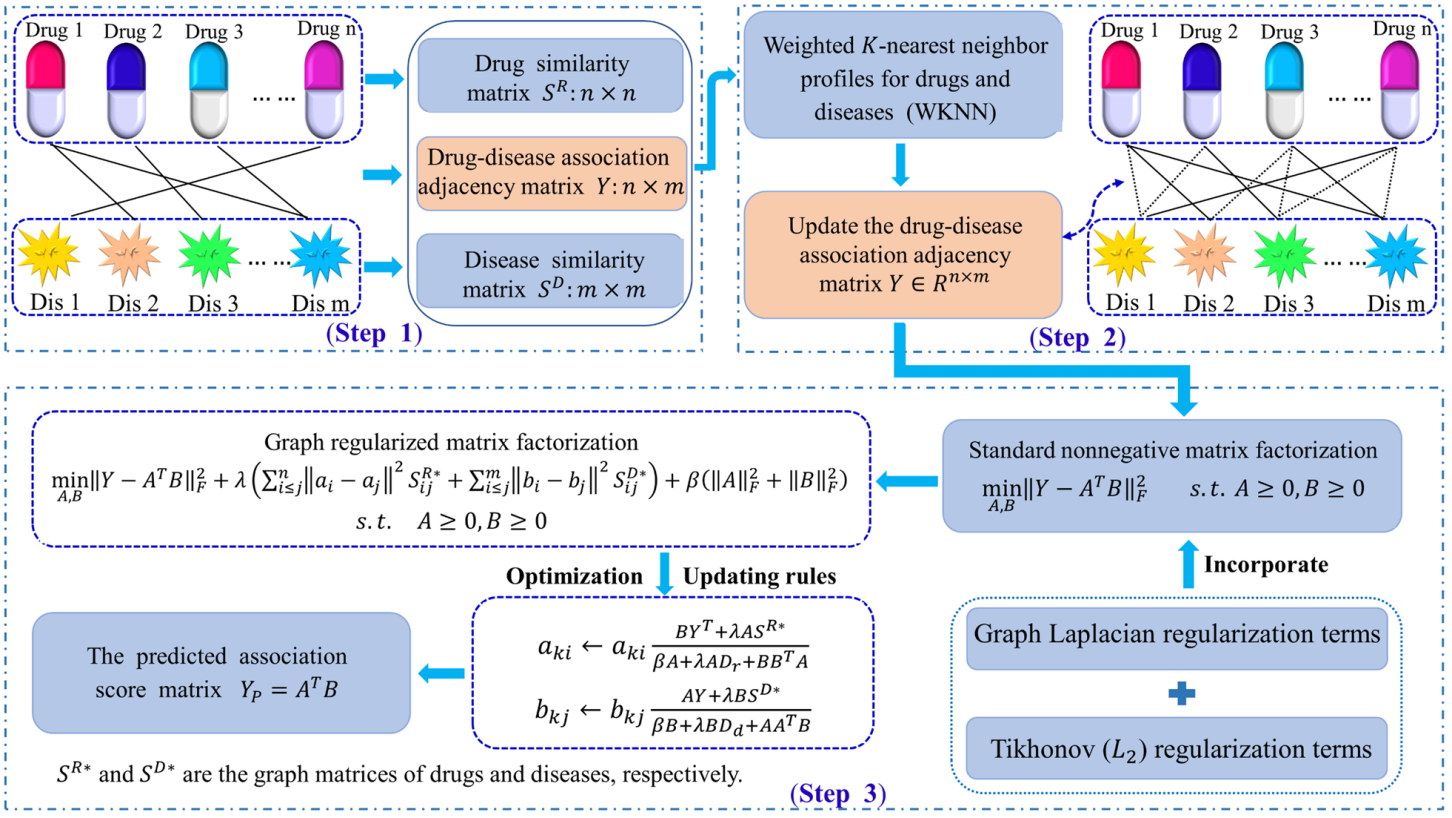
Flowchart of WNMFDDA for inferring the potential drug-disease associations

To identify potential associations between drug and disease, we propose a new computational model named WNMFDDA. The proposed method mainly process (See Fig. 1) contains three steps: (i) We measure the drug similarity and disease similarity based on chemical structures of drugs and medical description information of diseases, respectively. (ii) To extend WNMFDDA to predicting novel diseases and drugs, the adjacency matrix of drug with disease is reformulated based on weighted K-nearest neighbor profiles of drug and disease. (iii) Graph regularized collaborative matrix factorization is performed on the updated adjacency matrix to obtain the final score matrix.

### Datasets

The dataset (Fdataset) used in this work was obtained from Gottlieb et al. [19], which is comprised multiple data sources, and is considered as the golden standard datasets for predicting potential associations between drug and disease. After deleting the duplicate association pairs, a total of 1933 experimentally verified associations between 593 drugs and 313 diseases are collected for prediction. Diseases and drugs are obtained from the Online Mendelian Inheritance in Man (OMIM) database [34] and DrugBank database [35], respectively. Here, we construct the drug-disease association adjacency matrix ${Y}^{n\times m}$ based on the known associations, $n$ is the number of drugs and $m$ is the number of diseases. Let $R=\left\{{r}_{1},{r}_{2},\cdots ,{r}_{n}\right\}$ and $D=\left\{{d}_{1},{d}_{2},\cdots ,{d}_{m}\right\}$ represent the set of $n$ drugs and $m$ diseases. In the original adjacency matrix ${Y\in R}^{n\times m}$, the value of $Y(i,j)$ is set 1 if drug ${r}_{i}$ relates with disease ${d}_{j}$, otherwise it is 0. Finally, the original adjacency matrix ${Y\in R}^{593\times 313}$, the drug similarity matrix and disease similarity matrix are used to identify the associations of drugs with diseases based on WNMFDDA.

### Similarity for drugs and diseases

In this work, the drug similarity matrix is denoted by ${S}^{R}\in {R}^{593\times 593}$. we calculate the drug-drug similarity using the Chemical Development Kit (CDK) [36] based on Simplified Molecular Input Line Entry Specification (SMILES) chemical structures [37], and the Tanimoto score of their 2D chemical fingerprints is used as representing the pair of drug similarity [38].

The disease similarity matrix is denoted by ${S}^{D}\in {R}^{313\times 313}$. The similarities between diseases are derived from MimMiner [39], which measures the pairwise disease semantic similarity through text mining based on the medical description information in the OMIM database [34].

### Weighted graph regularized collaborative non-negative matrix factorization for predicting drug-disease associations

#### Reformulate association adjacency matrix of drug with disease

Due to many of non-interactions of drugs or diseases in the original adjacency matrix (i.e. their values are 0 in $Y$) that could be potential true interactions, which may lead to poor performance in predicting the potential drug-disease associations. In order to solve the above mentioned problem, we perform weighted $K$-nearest neighbor (WKNN) profiles to construct novel interaction profiles of drug and disease.

For each drug ${r}_{p}$, we sort all other drugs in descending order according to their similarities with ${r}_{p}$. Then, the new interaction profile of drug ${r}_{p}$ is obtained according to its $K$-nearest known drugs (each drug has at least one confirmed association), and their corresponding $K$ interaction profiles are as follows:

1 $${Y}_{r}\left({r}_{p}\right)=\frac{1}{{\sum }_{1\le i\le K}{S}^{R}({r}_{i,}{r}_{p})}{\sum }_{i=1}^{K}{w}_{i}Y({r}_{i})$$

where

2 $${w}_{i}={a}^{i-1}*{S}^{R}({r}_{i,}{r}_{p})$$

$a\in \left[\mathrm{0,1}\right]$ $\mathrm{is a decay term}$. ${w}_{i}$ is a weight coefficient, it means that the more similar ${r}_{i}$ to ${r}_{p}$, the larger weight is assigned. $Y\left({r}_{i}\right)=({Y}_{i1},{Y}_{i2},\cdots ,{Y}_{im})$ denotes the interaction profile for drug ${r}_{i}$, which is the $ith$ row vector of adjacency matrix $Y$.

Similar to drugs, for each disease ${d}_{q}$, the new interaction profiles of disease ${d}_{q}$ can be calculated as follows:

3 $${Y}_{d}\left({d}_{q}\right)=\frac{1}{{\sum }_{1\le j\le K}{S}^{D}({d}_{j,}{d}_{q})}{\sum }_{j=1}^{K}{w}_{j}Y({d}_{j})$$

4 $${w}_{j}={a}^{j-1}*{S}^{D}({d}_{j,}{d}_{q})$$

where, ${w}_{i}$ is a weight coefficient. $Y({d}_{j})=({Y}_{1j},{Y}_{2j},\cdots ,{Y}_{nj})$ represents the interaction profile for disease ${d}_{j}$, which is the $jth$ column vector of adjacency matrix $Y$.

Thereafter, we merge the new interaction profiles of drug and disease by ${Y}_{rd}=({Y}_{r}+{Y}_{d})/2$. Finally, the original adjacency matrix $Y$ is updated by replacing ${Y}_{ij}=0$ with related likelihood score as follows:

5 $$Y=\mathrm{max}(Y,{Y}_{rd})$$

#### The model of WNMFDDA

Non-negative matrix factorization (NMF) is one of the most popular multidimensional data processing tools in research fields such as bioinformatics and pattern recognition [40–42]. The purpose of NMF is to decompose a nonnegative matrix $Y$ into two low-dimensional nonnegative matrices, and makes their product approximation to the original matrix $Y$. Therefore, for drug-disease adjacency matrix ${Y}^{n\times m}$, it can be decomposed into two low-rank feature matrices, ${A}^{k\times n}$ and ${B}^{k\times m}$, and $Y\cong {A}^{T}B(k\le \mathrm{min}(n,m))$. The objective function for predicting drug-disease associations can be mathematically formulated as follows:

6 $$\underset{A,\mathit{ B}}{\mathrm{min}}{\Vert Y-{A}^{T}B\Vert }_{F}^{2} s.t. A\ge 0, B\ge 0$$

where ${\Vert \bullet \Vert }_{F}$ denotes the Frobenius norm. To enhance generalization capability and solve the problem that the standard NMF in formula (6) fails to discover the intrinsic geometrical of drug space and disease space, we introduce Laplacian regularization to constrain nonnegative matrix factorization which can ensure that close drugs or diseases are sufficiently close to each other in corresponding latent feature space. The optimization problem can be written as follows:

7 $$\underset{A,\mathit{ B}}{\mathrm{min}}{\Vert Y-{A}^{T}B\Vert }_{F}^{2}+\lambda \left(\sum_{i\le j}^{n}{\Vert {a}_{i}-{a}_{j}\Vert }^{2}{S}_{ij}^{R}+\sum_{i\le j}^{m}{\Vert {b}_{i}-{b}_{j}\Vert }^{2}{S}_{ij}^{D}\right)s.t. A\ge 0, B\ge 0$$

where ${R}_{1}=\sum_{i\le j}^{n}{\Vert {a}_{i}-{a}_{j}\Vert }^{2}{S}_{ij}^{R}$ and ${R}_{2}=\sum_{i\le j}^{m}{\Vert {b}_{i}-{b}_{j}\Vert }^{2}{S}_{ij}^{D}$ are the Laplacian regularization terms. ${a}_{i}$ and ${b}_{i}$ are $ith$ column of matrices $A$ and $B$, respectively. $\lambda$ is the regularization parameter.

Recent studies on manifold learning theory and spectral graph theory have shown that the local geometric structure and topological structure of original data points can be leaved unchanged by the $p$-nearest neighbor graph when these points are mapped from high-dimensional space to low-dimensional space [43, 44]. In addition, drugs and diseases in the same cluster are more possible to have similar characteristics, and the sparse similarity matrix has been effectively applied to graph regularization [45]. As a graph clustering method, $p$-nearest neighbor is used to construct the graphs (${S}^{R*}$ and ${S}^{D*}$) for drug space and disease space. Therefore, we can obtain the following weight matrix ${W}^{R}$ of drug according to the drug similarity matrix ${S}^{R}$:

8 $${W}_{ij}^{R}=\left\{\begin{array}{c} 1 i\in {N}_{p}\left({r}_{j}\right)\&j\in {N}_{p}\left({r}_{i}\right)\\ 0 i\notin {N}_{p}\left({r}_{j}\right)\&j\notin {N}_{p}\left({r}_{i}\right)\\ 0.5 otherwise\end{array}\right.$$

Here, ${N}_{p}\left({r}_{i}\right)$ and ${N}_{p}\left({r}_{j}\right)$ represent the sets of $p$-nearest neighbors of drug ${r}_{i}$ and drug ${r}_{j}$. Then, the graph matrix ${S}^{R*}$ for drugs is defined as follows:

9 $${\forall i, j {S}_{ij}^{R*}={S}_{ij}^{R}W}_{ij}^{R}$$

Similarly, based on the disease similarity matrix ${S}^{D}$, the graph matrix ${S}^{D*}$ for diseases is determined by:

10 $${\forall i, j {S}_{ij}^{D*}={S}_{ij}^{D}W}_{ij}^{D}$$

Then, the optimization problem is formularized as follows:

11 $$\underset{A,\mathit{ B}}{\mathrm{min}}{\Vert Y-{A}^{T}B\Vert }_{F}^{2}+\lambda \left(\sum_{i\le j}^{n}{\Vert {a}_{i}-{a}_{j}\Vert }^{2}{S}_{ij}^{R*}+\sum_{i\le j}^{m}{\Vert {b}_{i}-{b}_{j}\Vert }^{2}{S}_{ij}^{D*}\right) s.t. A\ge 0, B\ge 0$$

where ${R}_{1}^{*}=\sum_{i\le j}^{n}{\Vert {a}_{i}-{a}_{j}\Vert }^{2}{S}_{ij}^{R*}$ and ${R}_{2}^{*}=\sum_{i\le j}^{m}{\Vert {b}_{i}-{b}_{j}\Vert }^{2}{S}_{ij}^{D*}$ are the graph Laplacian regularization terms. In order to avoid overfitting and guarantee the $A$ and $B$ smoothness, Tikhonov (${L}_{2}$) regularization terms are incorporated into the Eq. (11) [46]. Finally, the optimization problem of WNMFDDA can be transformed into:

12 $$\underset{A,\mathit{ B}}{\mathrm{min}}{\Vert Y-{A}^{T}B\Vert }_{F}^{2}+\lambda \left(\sum_{i\le j}^{n}{\Vert {a}_{i}-{a}_{j}\Vert }^{2}{S}_{ij}^{R*}+\sum_{i\le j}^{m}{\Vert {b}_{i}-{b}_{j}\Vert }^{2}{S}_{ij}^{D*}\right)+\beta \left({\Vert A\Vert }_{F}^{2}+{\Vert B\Vert }_{F}^{2}\right) s.t. A\ge 0, B\ge 0$$

and

13 $$\sum_{i\le j}^{n}{\Vert {a}_{i}-{a}_{j}\Vert }^{2}{S}_{ij}^{R*}=\sum_{j=1}^{n}{a}_{j}^{T}{a}_{j}\sum_{i,j=1}^{n}{S}_{ij}^{R*}-\sum_{i,j=1}^{n}{a}_{i}^{T}{a}_{j}{S}_{ij}^{R*}=Tr\left(A{D}_{r}{A}^{T}\right)-Tr\left(A{S}^{R*}{A}^{T}\right)=Tr\left(A{L}_{r}{A}^{T}\right)$$

14 $$\sum_{i\le j}^{m}{\Vert {b}_{i}-{b}_{j}\Vert }^{2}{S}_{ij}^{D*}=Tr\left(B{D}_{d}{B}^{T}\right)-Tr\left(B{S}^{D*}{B}^{T}\right)=Tr\left(B{L}_{d}{B}^{T}\right)$$

where $\beta$ is the regularization parameter. $Tr\left(\bullet \right)$ is the trace of a matrix. ${D}_{r}=\sum_{i=1}^{n}{S}_{ij}^{R*}$ and ${D}_{d}=\sum_{i=1}^{m}{S}_{ij}^{D*}$ are the diagonal matrices; ${L}_{r}={D}_{r}-{S}^{R*}$ and ${L}_{d}={D}_{d}-{S}^{D*}$ denote the graph Laplacian matrices with respect to ${S}^{R*}$ and ${S}^{D*}$ [47]. The Eq. (12) can be rewritten as:

15 $$\underset{A,\mathit{ B}}{\mathrm{min}}{\Vert Y-{A}^{T}B\Vert }_{F}^{2} +\lambda \left(\sum_{i\le j}^{n}{\Vert {a}_{i}-{a}_{j}\Vert }^{2}{S}_{ij}^{R*}+\sum_{i\le j}^{m}{\Vert {b}_{i}-{b}_{j}\Vert }^{2}{S}_{ij}^{D*}\right)+\beta \left({\Vert A\Vert }_{F}^{2}+{\Vert B\Vert }_{F}^{2}\right)=Tr\left(Y{Y}^{T}\right)-2Tr\left(Y{B}^{T}A\right)+Tr\left({A}^{T}B{B}^{T}A\right)+\lambda Tr\left(A{L}_{r}{A}^{T}\right)+\lambda Tr\left(B{L}_{d}{B}^{T}\right)+\beta Tr\left(A{A}^{T}\right)+\beta Tr\left(B{B}^{T}\right)$$

#### Optimization algorithm

In this work, the optimization problem of objective function Eq. (15) is solved by using Lagrange multipliers method. We introduce Lagrange multipliers $\Phi =\{{\phi }_{ki}\}$ and $\Psi =\{{\psi }_{kj}\}$ to constrain ${a}_{ki}\ge 0$ and ${b}_{kj}\ge 0$, respectively. The corresponding Lagrange function ${\mathcal{L}}_{f}$ of Eq. (15) is represented as follows:

16 $${\mathcal{L}}_{f}=Tr\left(Y{Y}^{T}\right)-2Tr\left(Y{B}^{T}A\right)+Tr\left({A}^{T}B{B}^{T}A\right)+\lambda Tr\left(A{L}_{r}{A}^{T}\right) +\lambda Tr\left(B{L}_{d}{B}^{T}\right)+\beta Tr\left(A{A}^{T}\right)+\beta Tr\left(B{B}^{T}\right)+Tr\left(\Phi {A}^{T}\right)+Tr\left(\Psi {B}^{T}\right)$$

The partial derivatives of ${\mathcal{L}}_{f}$ to $A$ and $B$ are as follows:

17 $$\frac{\partial {\mathcal{L}}_{f}}{\partial A}=-2B{Y}^{T}+2B{B}^{T}A+2\lambda A{L}_{r}+2\beta A+\Phi $$

18 $$\frac{\partial {\mathcal{L}}_{f}}{\partial B}=-2AY+2A{A}^{T}B+2\lambda B{L}_{d}+2\beta B+\Psi $$

The Karush–Kuhn–Tucker (KKT) constraint conditions ${\phi }_{ki}{a}_{ki}=0$ and ${\psi }_{kj}{b}_{kj}=0$ are used in the following equations for ${a}_{ki}$ and ${b}_{kj}$ [48]:

19 $$-{\left(B{Y}^{T}\right)}_{ki}{a}_{ki}+{\left(B{B}^{T}A\right)}_{ki}{a}_{ki}+{\left[\lambda A\left({D}_{r}-{S}^{R*}\right)\right]}_{ki}{a}_{ki}+{\left(\beta A\right)}_{ki}{a}_{ki}=0$$

20 $$-{\left(AY\right)}_{kj}{b}_{kj}+{\left(A{A}^{T}B\right)}_{kj}{b}_{kj}+{\left[\lambda B\left({D}_{d}-{S}^{D*}\right)\right]}_{kj}{b}_{kj}+{(\beta B)}_{kj}{b}_{kj}=0$$

Finally, the updating rules for ${a}_{ki}$ and ${b}_{kj}$ can be determined as follows:

21 $${a}_{ki}\leftarrow {a}_{ki}\frac{B{Y}^{T}+\lambda A{S}^{R*}}{\beta A+\lambda A{D}_{r}+B{B}^{T}A}$$

22 $${b}_{kj}\leftarrow {b}_{kj}\frac{AY+\lambda B{S}^{D*}}{\beta B+\lambda B{D}_{d}+A{A}^{T}B}$$

We update the matrices $A$ and $B$ with Eq. (21) and Eq. (22) until convergence. The predicted association score matrix for drug-disease pairs is obtained by ${Y}_{P}={A}^{T}B$. Then, we prioritize the disease-associated drugs (or drug-associated diseases) on the basis of correlation scores in matrix ${Y}_{P}$. Generally, the higher the drug-disease pair score, the more likely they are to be related. The whole algorithm of WNMFDDA is exhibited in Table 1.

**Table 1:**
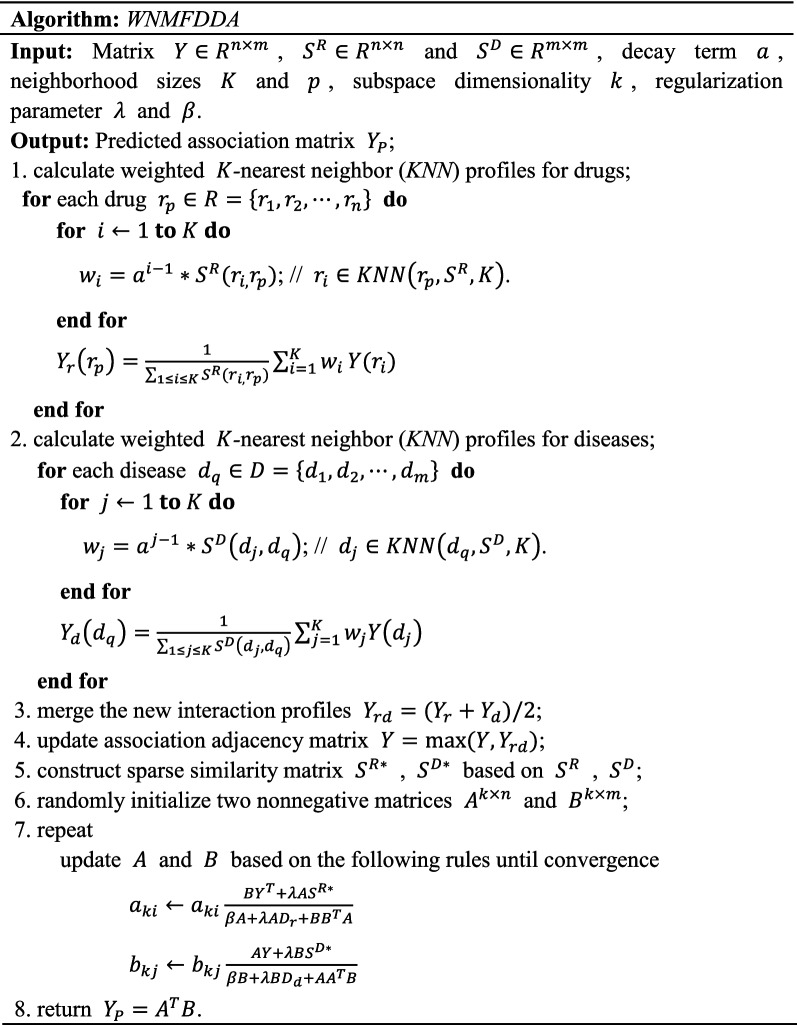
The algorithm for predicting drug-disease associations

## Results and discussion

### Experimental settings

To systematically assess the ability of WNMFDDA in predicting potential associations of drugs with diseases, we conduct ten-fold cross validation (10-CV) experiments based on known drug-disease associations. In the golden dataset, 1933 known associations of drugs with diseases are randomly divided into ten roughly equal parts, while the other unconfirmed pairs are regarded as candidate associations. In each cross validation, each part is served as a test set in turn, and the remaining parts are treated as the training set.

AUC is widely applied for assessing the prediction models [49]. Since the known drug-disease associations are much less than unknown associations between them, the sensitivity (Sen., also known as recall) and Precision (Pre.) are computed as the evaluation metric. In addition, other classification metrics, accuracy (Acc.) and F1-Score, are also used widely [50].

23 $$Sen.=\frac{TP}{TP+FN}$$

24 $$Pre.=\frac{TP}{TP+Fp}$$

25 $$Acc.=\frac{TN+TP}{TN+TP+FN+Fp}$$

26 $$F1-Score=\frac{2\times Pre.\times Sen.}{Pre.+Sen.}$$

In this work, the influence of parameters on WNMFDDA has been analyzed by applying Fdataset. We used grid search to determine the parameter combinations. WNMFDDA has six parameters and their values are considered from the following ranges: decay term $a\in \left\{0.1, 0.2,\cdots ,1\right\}$, neighborhood size $K$ is chosen from $\left\{1, 2,\cdots ,10\right\}$, subspace dimensionality $k\in \left\{60, 80, 100,\cdots , 200\right\}$, regularization coefficients $\lambda \in \left\{0.02, 0.2, 1, 2\right\}$ and $\beta \in \left\{0.002, 0.02, 0.2, 1\right\}$. At the same time, we set $p=5$ to construct the graphs for drug space and disease space according to [43] and [51]. The final optimal parameter combinations are $K=5$, $a=0.5$, $k=160$, $\lambda =1$ and $\beta =0.02$, which are determined based on AUC values under 10-CV experiments. Meanwhile, we used the best parameter values that recommended by the corresponding authors in compared methods.

### Performance evaluation

**Fig. 2:**
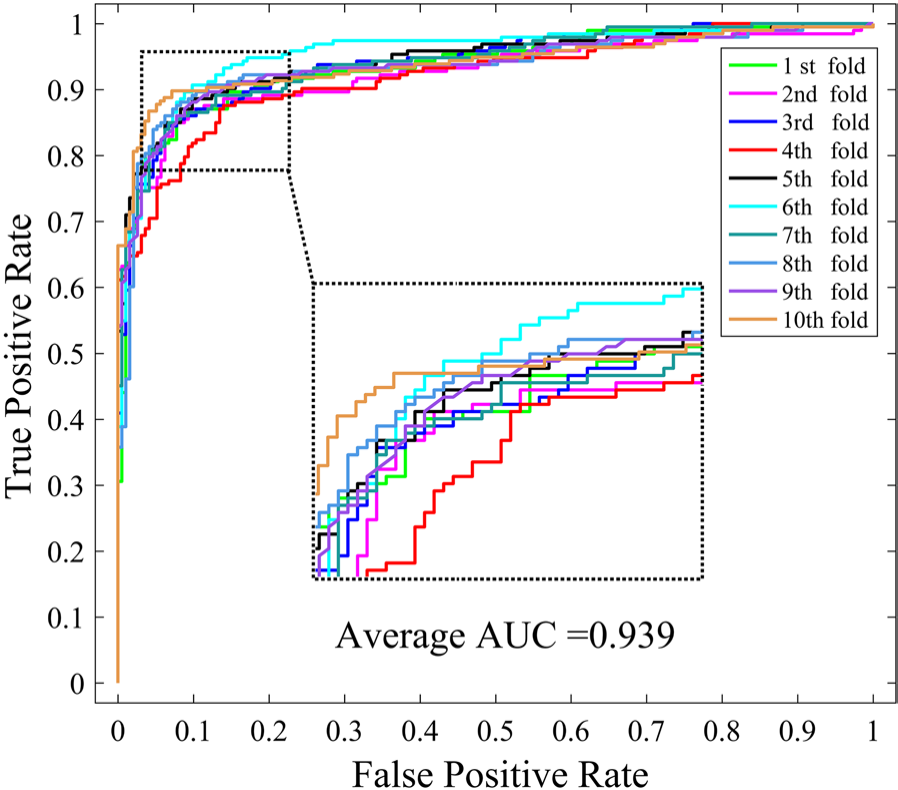
The ROC curves of WNMFDDA on Fdataset under ten-fold cross validation

In this study, ten-fold cross validation was introduced to assess the performance of WNMFDDA. we conduct 10-CV on the Fdataset to compare it with four classical models, including DDRS [27], MBiRW [24], HGBI [23] and DrugNet [22]. As shown in Fig. 2, the AUC value achieved by WNMFDDA is 0.939. The AUC values of WNMFDDA and the other four competing approaches on Fdataset are displayed in Table 2. Specifically, the AUC values of WNMFDDA, DDRS, MBiRW, HGBI and DrugNet are 0.939, 0.930, 0.917, 0.829 and 0.778, respectively. The performance of WNMFDDA method outperforms the compared computational approaches, DDRS, MBiRW, HGBI and DrugNet.

**Table 2:** The average AUC values of WNMFDDA and related methods on Fdataset

Methods	DDRS	MBiRW	HGBI	DrugNet	WNMFDDA
AUC	0.930	0.917	0.829	0.778	0.939

**Fig. 3:**
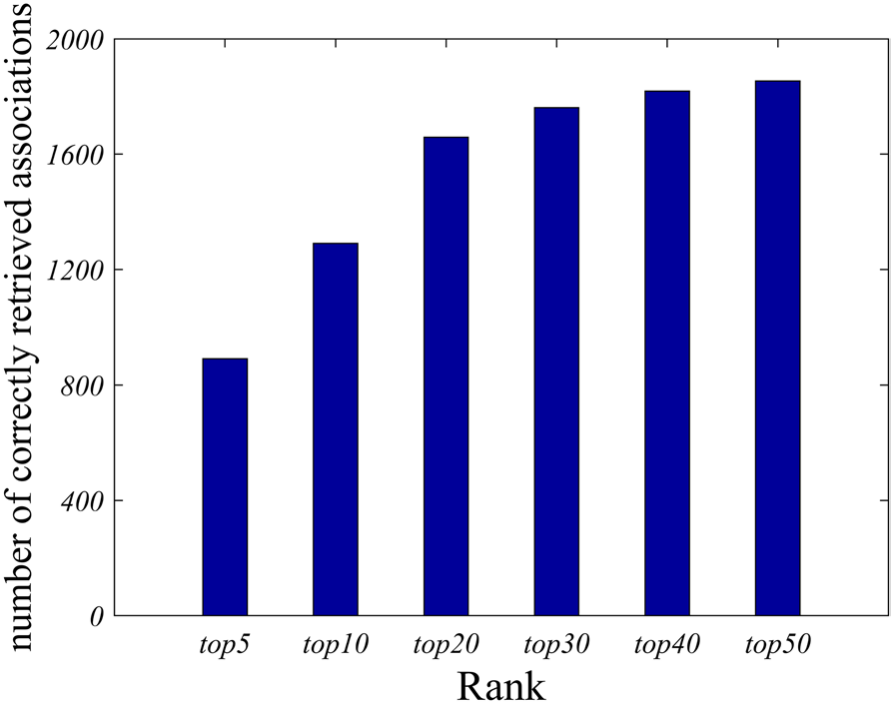
The number of correctly retrieved drug-disease associations for various ranking thresholds

In practice, the predicted top-ranked results are more important than other parts. In this study, the numbers of correctly retrieved true associations between drug and disease from different top portions were counted when all known associations are regarded as the training set. In generally, the method is considered as more reliable if more true associations are discovered on the top portions. At different thresholds, the number of true associations correctly predicted by WNMFDDA are shown in Fig. 3. For example, at the top 20 and 40 of predicted candidate drugs, WNMFDDA correctly identified 1651 (85.41%) and 1819 (94.10%) true associations from all the 1933 known associations, respectively. The experimental results suggest that our model has higher accuracy and lower false positive rate in identifying potential drug-disease associations.

In addition, considering the fact that the known and unknown associations between them are serious imbalance, several classification metrics (i.e. Sen., Pre., Acc. and F1-Score) are calculated at different specificity (Spe.), and are used as evaluation indicators. As shown in Table 3, the average Sen, Pre, Acc and F1-Score are 86.91%, 89.79%, 88.51% and 88.31%, respectively, when Spe is 90%. This result further illustrates that our method is reliable.

**Table 3:** The ten-fold cross validation results achieved by WNMFDDA on Fdataset

Test set	Sen.(%)	Pre.(%)	Acc.(%)	F1-Score(%)
1 2 3 4 5 6 7 8 9 10 Average	86.53 87.05 86.01 81.35 87.05 89.64 86.53 88.60 86.53 89.80 86.91 ± 2.38	89.78 89.84 89.73 89.20 89.84 90.10 89.78 90.00 89.78 89.80 89.79 ± 0.24	88.34 88.60 88.08 85.75 88.60 89.90 88.34 89.38 88.34 89.80 88.51 ± 1.17	88.13 88.42 87.83 85.09 88.42 89.87 88.13 89.30 88.13 89.80 88.31 ± 1.35

### Case studies

In this section, to further test the predictive performance of WNMFDDA, we conduct two types of case studies on two drugs and two diseases, respectively. The first type of case study was performed on Doxorubicin drug and Obesity. During the experiment, all known associations on the Fdataset are utilized to train prediction model of WNMFDDA. For Doxorubicin, the top-15 candidate diseases related with Doxorubicin are obtained according to their predicted association scores. Then, we validate these candidate diseases based on the other public biological database: Comparative Toxicogenomics Database (CTD) [52], which provides newly experimentally verified associations between drugs and diseases. Table 4 lists the top-15 predicted candidate diseases for Doxorubicin, 12 out of the top-15 are confirmed by CTD to be associated with Doxorubicin. For example, Doxorubicin, originally indicated for Acute Leukemia, is predicted to treat stomach cancer and confirmed by CTD. As shown in Table 5, 13 out of the top-15 predicted drugs are confirmed by CTD to be associated with Obesity.

**Table 4:** The top-15 candidate diseases associated with Doxorubicin are predicted by GWMFDDA based on known associations in Fdataset

Drug	Rank	Diseases	Evidences	Rank	Diseases	Evidences
*Doxorubicin*	1	Turcot syndrome	CTD	9	Urinary Bladder Neoplasms	CTD
	2	Lymphoblastic Leukemia, Acute, with Lymphomatous Features	unconfirmed	10	Neuroblastoma	CTD
	3	Breast Neoplasms	CTD	11	Testicular Germ Cell Tumor	CTD
	4	Hodgkin Disease	CTD	12	Multiple Myeloma	CTD
	5	Leukemia, Myeloid, Acute	CTD	13	Carcinoma, Small Cell	CTD
	6	Dohle Bodies And Leukemia	unconfirmed	14	Stomach Neoplasms	CTD
	7	Rhabdomyosarcoma 2	CTD	15	Reticulum Cell Sarcoma	unconfirmed
	8	Osteosarcoma	CTD

**Table 5:** The top-15 candidate drugs associated with Obesity are predicted by GWMFDDA based on known associations in Fdataset

Disease		Rank	Drugs		Evidences		Rank	Drugs			Evidences
		1	Benzphetamine		CTD		9	Bupropion			CTD
		2	Phentermine		CTD		10	Amphetamine			CTD
		3	Phenylpropanolamine		CTD		11	Pseudoephedrine		unconfirmed	
*Obesity*		4	Sibutramine		CTD		12	Dextroamphetamine			CTD
		5	Metamfetamine		unconfirmed		13	Ephedrine			CTD
		6	Orlistat		CTD		14	Cimetidine			CTD
		7	Phendimetrazine		CTD		15	Topiramate			CTD
		8	Diethylpropion		CTD						

In order to illustrate the prediction capability of WNMFDDA on novel diseases /drugs without known associated drugs/diseases, we selected Amiodarone drug and Asthma disease to perform the second type of case study. For drug Amiodarone, before training the model, all known associations with Amiodarone are removed from the original dataset. Then, we sort all the 313 diseases in descending order according to the correlation scores, and verify the top-15 diseases in the CTD. As shown in Table 6, 12 out of the top-15 drug-disease associations predicted by WNMFDDA are confirmed in the CTD. Similarly, all known associations with Asthma are hidden from the original dataset when we carry out case study to Asthma. The top-15 inferred candidate drugs are displayed in Table 7, 13 out of 15 are verified to be related with the Asthma by CTD. These results further suggest that WNMFDDA is a useful predictor to infer potential associations of diseases with drugs.

**Table 6:** The top-15 candidate diseases associated with Amiodarone are predicted by GWMFDDA after removing all known associations with Amiodarone based on the Fdataset

Drug	Rank	Diseases	Evidences	Rank	Diseases	Evidences
*Amiodarone*	1	Breast Neoplasms	CTD	9	Hodgkin Disease	CTD
	2	Lymphoblastic Leukemia, Acute, with Lymphomatous Features	CTD	10	Osteosarcoma	CTD
	3	Leukemia, Myeloid, Acute	CTD	11	Inclusion Body Myopathy With Early-Onset Paget Disease And Frontotemporal Dementia	CTD
	4	Turcot Syndrome	Unconfirmed	12	Urinary Bladder Neoplasms	CTD
	5	Dohle Bodies and Leukemia	Unconfirmed	13	Lung Neoplasms	CTD
	6	Hajdu-Cheney Syndrome	Unconfirmed	14	Carcinoma, Small Cell	CTD
	7	Multiple Myeloma	CTD	15	Fibrous Dysplasia, Polyostotic	CTD
	8	Osteoporosis	CTD

**Table 7:** The top-15 candidate drugs associated with Asthma are predicted by GWMFDDA after removing all known associations with Asthma based on the Fdataset

Disease	Rank	Drugs	Evidences	Rank	Drugs	Evidences
*Asthma*	1	Cromoglicic acid	Unconfirmed	9	Triamcinolone	CTD
	2	Ciprofloxacin	CTD	10	Montelukast	CTD
	3	Budesonide	CTD	11	Beclomethasone	CTD
	4	Pirbuterol	CTD	12	Moxifloxacin	CTD
	5	Salbutamol	CTD	13	Nedocromil	CTD
	6	Zileuton	CTD	14	Formoterol	CTD
	7	Prednisone	CTD	15	Orciprenaline	Unconfirmed
	8	Terbutaline	CTD			

### Validation on the other dataset

**Fig. 4:**
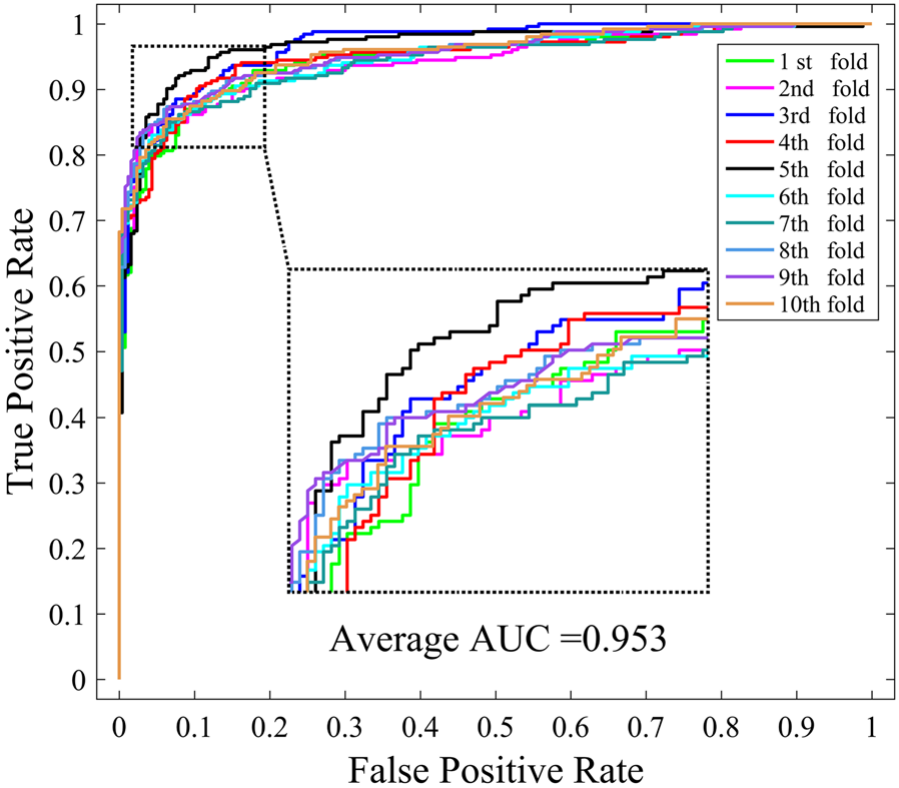
ROC curves of WNMFDDA on Cdataset under ten-fold cross validation

To further validate the robustness of WNMFDDA, we implement 10-CV to verify the prediction accuracy on the Cdataset. This dataset has been used in previous studies [24, 27], including 663 drugs, 409 diseases and 2532 verified drug-disease associations. These drugs and diseases are obtained from DrugBank database and OMIM database, respectively. The ROC curves of WNMFDDA on Cdataset are drawn in Fig. 4. The average AUC values of WNMFDDA and the compared methods are shown in Table 8. We can see that the average AUC value of WNMFDDA is 0.953, while DDRS, MBiRW, HGBI and DrugNet are 0.947, 0.933, 0.858 and 0.804, respectively. WNMFDDA achieves the best prediction performance. The superior experiment results on Cdataset also demonstrate that our proposed model is robust and effective in revealing potential associations between drug and disease.

**Table 8:** AUC values of WNMFDDA and related methods on Cdataset

Methods	DDRS	MBiRW	HGBI	DrugNet	WNMFDDA
AUC	0.947	0.933	0.858	0.804	0.953

## Conclusions

Identifying new indications for existing drugs is a promising alternative to drug development, which not only saves time and costs, but also reduces risks and expedites drug approval. In this work, a model based on weight non-negative matrix factorization, WNMFDDA, was proposed to predict potential drug-disease associations. Different from other traditional computational methods, WNMFDDA reformulate the adjacency association matrix based on weighted $K$ nearest neighbor profiles as a preprocessing step, which enables it to infer potential associations for novel diseases/drugs without any known associated with drugs/diseases. Meanwhile, graph regularized matrix factorization was used to calculate the association scores.

We conducted 10-CV on two datasets and case studies on Fdataset to verify the performance of our developed model. Comprehensive experimental results demonstrate that WNMFDDA outperforms other state-of-the-art approaches, and can effectively infer potential associations between drug and disease. We believe that WNMFDDA is helpful for relevant biomedical researchers in follow-up studies. However, WNMFDDA still has some limitations. Firstly, the number of experimental verified drug-disease associations used in this work is relatively sparse. Secondly, determining the optimal parameter combinations for different biological datasets is still a daunting task. Finally, how to reasonably incorporate more effective drug and disease features to enhance the performance of WNMFDDA deserves further research.

## Data Availability

The datasets that we collected in this work is freely available on https://github.com/meinengwang/WNMFDDA.
